# Tofacitinib Treatment in Patients With Active COVID-19 Infection

**DOI:** 10.7759/cureus.17957

**Published:** 2021-09-14

**Authors:** Abdulmajeed M Alajlan, Lama R AlZamil, Amal M Aseri

**Affiliations:** 1 Department of Dermatology, College of Medicine, King Saud University, Riyadh, SAU; 2 Department of Dermatology, College of Medicine, Qassim University, Buraidah, SAU

**Keywords:** tofacitinib, alopecia areata, jak inhibitor, covid19, jak1, jak3

## Abstract

Alopecia areata is a chronic autoimmune disorder attacking the hair follicle epithelium; hence, causing non-scarring hair loss. It has been found that Janus kinase 3 (JAK3) hyperactivity plays a key role in the pathogenesis of the disease. Tofacitinib is an effective JAK1 and JAK3 inhibitor that can block several cytokines such as IL-2, IL-7, and IL-6. Several studies have demonstrated the efficacy of oral tofacitinib in hair regrowth in alopecia areata patients. With the recent COVID-19 pandemic, it has been advised to withhold JAK inhibitors during the period of active infection due to possible immunosuppression. We herein report two cases of patients with alopecia universalis who continued to use tofacitinib during their active COVID-19 infection and showed no deterioration in their course of illness.

## Introduction

Alopecia areata (AA) is a chronic autoimmune disorder attacking the hair follicle epithelium; hence, causing non-scarring hair loss. It most commonly involves the scalp. The prevalence of AA in Saudi Arabia, as estimated through a social-media-based questionnaire, is around 13.8% [1]. Genetic predisposition and autoimmunity play an important role in the pathogenesis of alopecia; however, other factors such as psychological distress can also trigger the disease. It has been found that Janus kinase 3 (JAK3) hyperactivity plays a key role in the pathogenesis of the disease [2]. Hence, the introduction of JAK inhibitors into the management of alopecia has made a great impact on alopecia-affected patients. Tofacitinib is an effective JAK1 and JAK3 inhibitor that can block several cytokines such as IL-2, IL-7, and IL-6 [3]. With the recent COVID-19 pandemic, it has been advised to withhold JAK inhibitors during the period of active infection due to possible immunosuppression [2]. We herein report two cases of patients with alopecia universalis who continued to use tofacitinib during their active COVID-19 infection and showed no deterioration in their course of illness.

## Case presentation

Case 1

**Figure 1:**
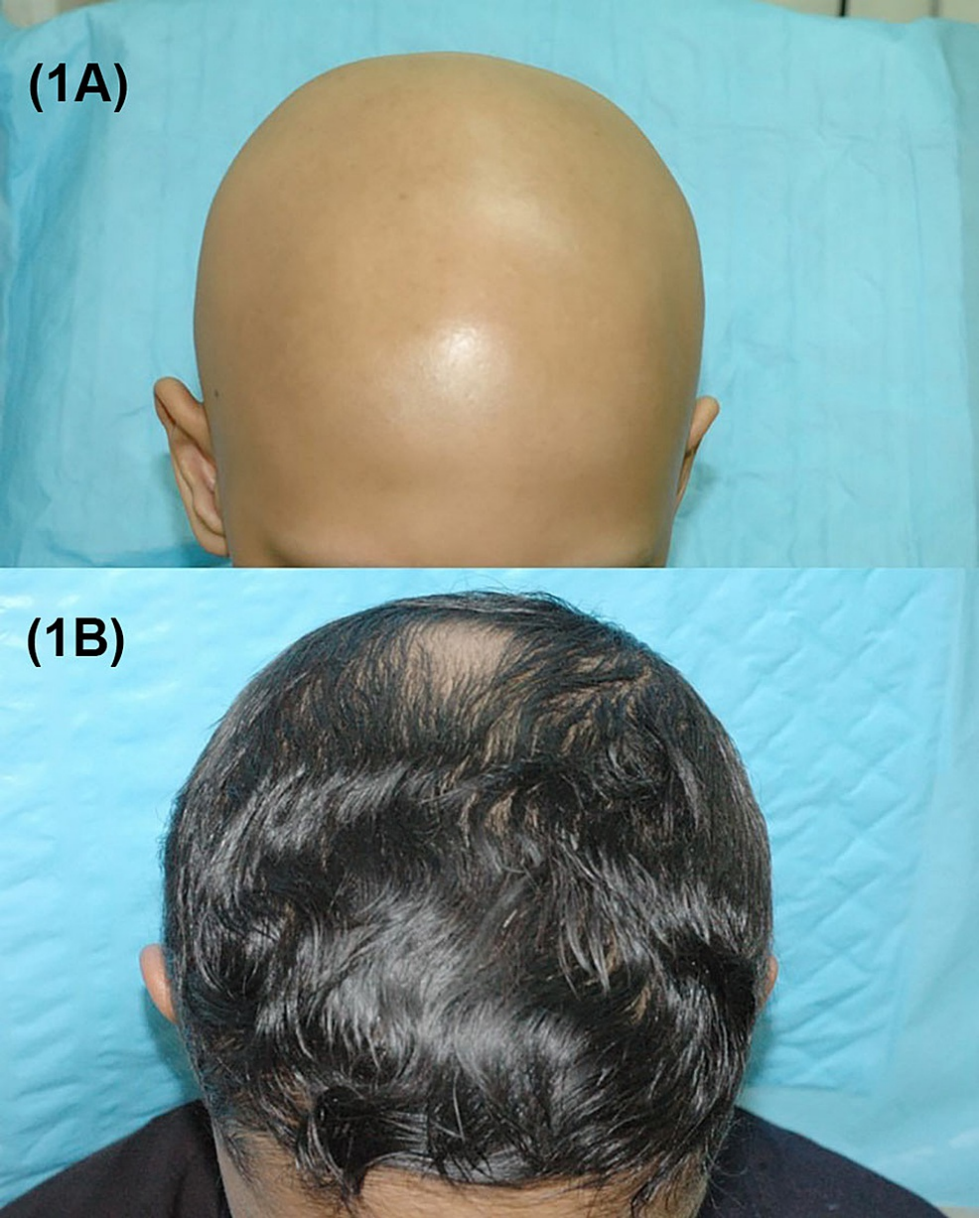
Alopecia universalis. (1A) Before oral tofacitinib treatment (1B) Two-year follow-up with almost complete hair regrowth

A 27-year-old Saudi female patient had alopecia universalis for 16 years and comorbid hypothyroidism, for which she was on thyroxine. Her condition started as alopecia areata and then progressed to alopecia universalis as she grew up. She had previously received various medications for the treatment of alopecia, such as oral prednisolone, methotrexate, topical minoxidil solution 5%, and intralesional Kenalog (ILK) injections. Then she was introduced to oral tofacitinib treatment. She used tofacitinib for two years and reported almost complete hair regrowth with no signs of disease activity since then (Figures 1A, 1B). In July 2020, she tested positive for COVID-19 infection while using tofacitinib 5 mg twice a day and prednisolone 7.5 mg once a day. During her illness with COVID-19, she had only experienced mild symptoms of body ache, anosmia, and ageusia. She had no symptoms of fever, cough, or shortness of breath. She was self-quarantined at home and did not require hospital admission. During her infection, she continued to take tofacitinib despite the medical advice of withholding the medication throughout the period of her active infection. She did not notice any hair fall throughout and after her illness with COVID-19. Rather she reported improvement in regards to hair fall. Recently, i.e., post five months of getting infected with COVID-19, she noticed less hair density in some parts. However, this did not seem to upset her. Overall, she was satisfied with the result of the treatment.

Case 2

A 33-year-old Saudi female patient had alopecia universalis for 23 years. Her condition, which started at the age of 10, began as hair loss mainly at the occipital area and gradually progressed to alopecia universalis. She had previously used oral prednisolone and ILK injections for managing her condition. However, no satisfactory improvement was noticed until she started using oral tofacitinib treatment two years ago. In November 2020, she tested positive for COVID-19 infection while using tofacitinib 5 mg twice daily and prednisolone 5 mg once daily. During her illness, she had only experienced mild symptoms of fever, headache, anosmia, and low appetite. She denied any cough or shortness of breath. She was self-quarantined at home and did not require hospital admission. Throughout the duration of her infection, she stopped taking the medication for the first four days but then resumed it by herself when her symptoms diminished. She did not notice any changes in hair fall throughout and after her illness with COVID-19.

## Discussion

The Janus kinase (JAK) family and its substrate, the signal transducer and activator of transcription (STAT) are a group of receptor-associated signaling molecules that are essential to the signal cascade through which cytokines exert and integrate their function. Cytokines are critical for host defense and immunoregulation. They also play a major role in the pathogenesis of various autoimmune diseases such as alopecia areata. The four enzymes of the JAK family include JAK1, JAK2, JAK3, and tyrosine kinase 2 (TYK2), which are the key components in initiating the intracellular signaling cascade of the JAK/STAT pathway [4]. Therefore, the critical function of JAKs in cytokine signaling has made them a target in the development of various drugs for the treatment of several autoimmune diseases and malignancies [3,4]. The JAK inhibitors are a class of drugs that block one or more of the JAK enzymes. Tofacitinib is an effective JAK1 and JAK3 inhibitor and, to a lesser extent, also a JAK2 inhibitor, which can block several cytokines. Tofacitinib efficiently blocks the common γ-chain cytokines, including IL-2, IL-7, IL-15, and IL-21, which signal through JAK3 and block JAK1, which subsequently results in inhibition of IL-6, IL-11, and IFN-γ [3,4]. As a result, tofacitinib impairs the differentiation of CD4+ T helper cells, interferes with the differentiation of IFN-γ producing Th1 cells, and blocks the generation of pathogenic Th17 cells through IL-23 signaling blockade [3,4]. Tofacitinib was first approved by the United States Food and Drug Administration (FDA) in late 2012 for the treatment of moderate-to-severe rheumatoid arthritis [5]. Several studies and case reports have demonstrated the efficacy of oral tofacitinib treatment in hair regrowth in alopecia areata patients [6].

With the recent COVID-19 pandemic, it has been advised to discontinue immunosuppressive medications, including tofacitinib, during active COVID-19 infection due to the possible alteration of the immune response by these drugs, which could worsen the course of the infection [2]. Conversely, it has been reported that a 33-year-old woman with ulcerative colitis (UC) who was controlled on tofacitinib 10 mg twice daily had acquired the COVID-19 infection, and she continued to use tofacitinib during her illness without interruption. Despite that, her respiratory symptoms resolved in five days, and she recovered completely in two weeks with no change to tofacitinib treatment [7]. In addition, other studies have investigated the use of tofacitinib and other JAK inhibitors on the outcomes of COVID-19 infection and have concluded that there is no statistically significant difference in the rate of hospitalization, admission to the ICU, and severe COVID-19 infection among tofacitinib-treated patients and other patients [8,2]. In fact, a recently published systematic review sheds light on the potential efficacy of JAK inhibitors and Type I interferons against the COVID-19 infection and its association with positive clinical outcomes in terms of hospitalization, ICU admission, and mortality rate [9]. Therefore, JAK inhibitors, particularly baricitinib, have been proposed as a potential therapy for COVID-19 by interrupting the virus’s integration into host cells via disruption of AP2-associated protein kinase-1 (AAK1) signaling [10].

## Conclusions

To conclude, in this report, we presented the cases of two healthy patients who acquired COVID-19 infection and recovered completely while continuing to use tofacitinib during their infection. This case report demonstrates that tofacitinib may not need to be held in patients with COVID-19 without severe illness.
